# Left atrial appendage closure devices: a contemporary review of techniques, tips and tricks

**DOI:** 10.3389/fcvm.2026.1767568

**Published:** 2026-04-15

**Authors:** Kamran Namjouyan, Hafeez Ul Hassan Virk, Affan Rizwan, Muzamil Khawaja, Mahboob Alam, Markus Strauss, Chayakrit Krittanawong

**Affiliations:** 1 Department of Cardiology, Geisinger Medical Center, Danville, PA, United States; 2 Division of Cardiovascular Disease, Department of Medicine, Albert Einstein Healthcare Network, Philadelphia, PA, United States; 3 Department of Internal Medicine, Baylor College of Medicine, Houston, TX, United States; 4 Department of Cardiology, Emory University, Atlanta, GA, United States; 5 Texas Heart Institute and Baylor College of Medicine, Houston, TX, United States; 6 Department of Cardiology I- Coronary and Peripheral Vascular Disease, Heart Failure Medicine, University Hospital Muenster, Cardiol, Muenster, Germany; 7 HumanX, Delaware City, DE, United States

**Keywords:** atrial fibrillation, DAPT, LAA closure, left atrial appendage closure (LAAC), Watchman device

## Abstract

Atrial fibrillation (AF) affects approximately 3%–5% of adults and is projected to double in prevalence by 2060, significantly increasing the burden of thromboembolic stroke. This risk is largely attributed to thrombus formation within the left atrial appendage (LAA), particularly in nonvalvular AF, where the LAA's trabeculated anatomy, diverse morphologies (e.g., chicken wing, windsock, cactus, cauliflower), and impaired contractility allows for blood stasis and thrombogenesis. As a result, the LAA has become a focus for stroke prevention strategies. This review demonstrates current evidence on LAA closure by discussing patient selection, anatomical feasibility, procedural workflow, imaging guidance (transesophageal echocardiography vs. intracardiac echocardiography), device platforms (Amplatzer Amulet, Watchman 2.5, Watchman FLX/FLX Pro), and complication profiles. P procedural success rates exceed 95%, with improving safety profiles. Nonetheless, adverse events such as pericardial effusion, device-related thrombus (DRT), peri-device leak (PDL), device embolization, and periprocedural stroke remain important considerations. Post-implant antithrombotic strategies are evolving beyond the traditional warfarin/aspirin → DAPT → SAPT pathway toward more individualized regimens, including simplified DOAC or antiplatelet-based approaches tailored to bleeding and thrombotic risk especially in complex scenarios like concurrent coronary stenting. Ongoing device innovations (e.g., FLX Pro, expanded size matrices, thromboresistant coatings), CT-led surveillance algorithms, and randomized studies of post-implant pharmacotherapy aim to reduce DRT/bleeding, harmonize follow-up, and expand indications. Collectively, these advances refine patient-centered LAA closure by aligning anatomic complexity, procedural technique, and pharmacology to improve stroke prevention and safety in AF.

## 1 Introduction

Atrial fibrillation (AF) is a prevalent cardiac arrhythmia, currently affecting approximately 3.2% of adults. Its prevalence is projected to double by 2060, driven by factors such as increased life expectancy and rising rates of obesity, hypertension, and diabetes. In the United States, an estimated 10.55 million adults about 4.48% of the adult population have been diagnosed with AF. If left undetected and untreated, AF can lead to serious complications such as ischemic stroke, heart failure, and cognitive decline (1, 2). Thromboembolic stroke prevention is one of the most critical components of AF management. Strokes associated with AF are typically more severe, leading to higher mortality rates, greater long-term disability, longer hospital stays, and a lower likelihood of discharge to home compared to strokes unrelated to AF (3). The left atrial appendage (LAA) holds significant clinical importance as it is the primary site of thrombus formation in patients with AF, responsible for over 90% of left atrial thrombi in cases of nonvalvular AF (4). This predisposes patients to cardioembolic complications, particularly ischemic stroke. The LAA's complex anatomy and trabeculated structure contribute to blood stasis, especially when its contractile function is compromised. Consequently, the LAA has become a central focus of stroke prevention strategies, including both anticoagulation (AC) therapy and device-based occlusion (5). This review aims to explore the anatomical and clinical significance of LAA in the context of AF -related stroke risk, and to critically examine the latest innovations in LAA closure techniques and comparing current literature. By evaluating emerging device-based strategies and procedural advancements, the article seeks to highlight how these developments are shaping contemporary stroke prevention paradigms and improving patient outcomes.

## 2 Morphology of LAA and pathophysiology of thrombus formation

The LAA is a finger-like, blind-ended muscular pouch that extends from the lateral wall of the left atrium. Embryologically distinct from the main chamber, the LAA retains unique structural and physiological characteristics. Functionally, the LAA serves multiple roles. It acts as a decompression chamber by helping to regulate left atrial pressure and enhance atrial compliance during periods of hemodynamic stress. Additionally, it is rich in atrial natriuretic peptide granules, which are released in response to atrial stretch and contribute to fluid and electrolyte balance (5–7).

The LAA plays a central role in thrombus formation due to blood stasis. This stasis is increased by the LAA's intricate, trabeculated structure and its diminished contractile function during AF. In the setting of AF, the loss of synchronized atrial contraction significantly reduces flow velocities within the LAA, creating conditions for blood stagnation and increasing the risk of thrombus development (4, 8). Additionally, the LAA's narrow orifice and highly variable morphology contribute to regions of low shear stress and prolonged blood residence time. This will then further facilitating clot formation. Patients with AF exhibit distinct prothrombotic conditions within the LAA, including the development of dense fibrin clots and delayed clot breakdown. These findings suggest that the LAA provides a unique intracardiac environment that promotes thrombosis more than other heart chambers. In nonvalvular AF, most thrombi originate in the LAA and it is considered the primary source of cardioembolic stroke (9, 10).

## 3 Anatomic evaluation for feasibility

**Figure 1:**
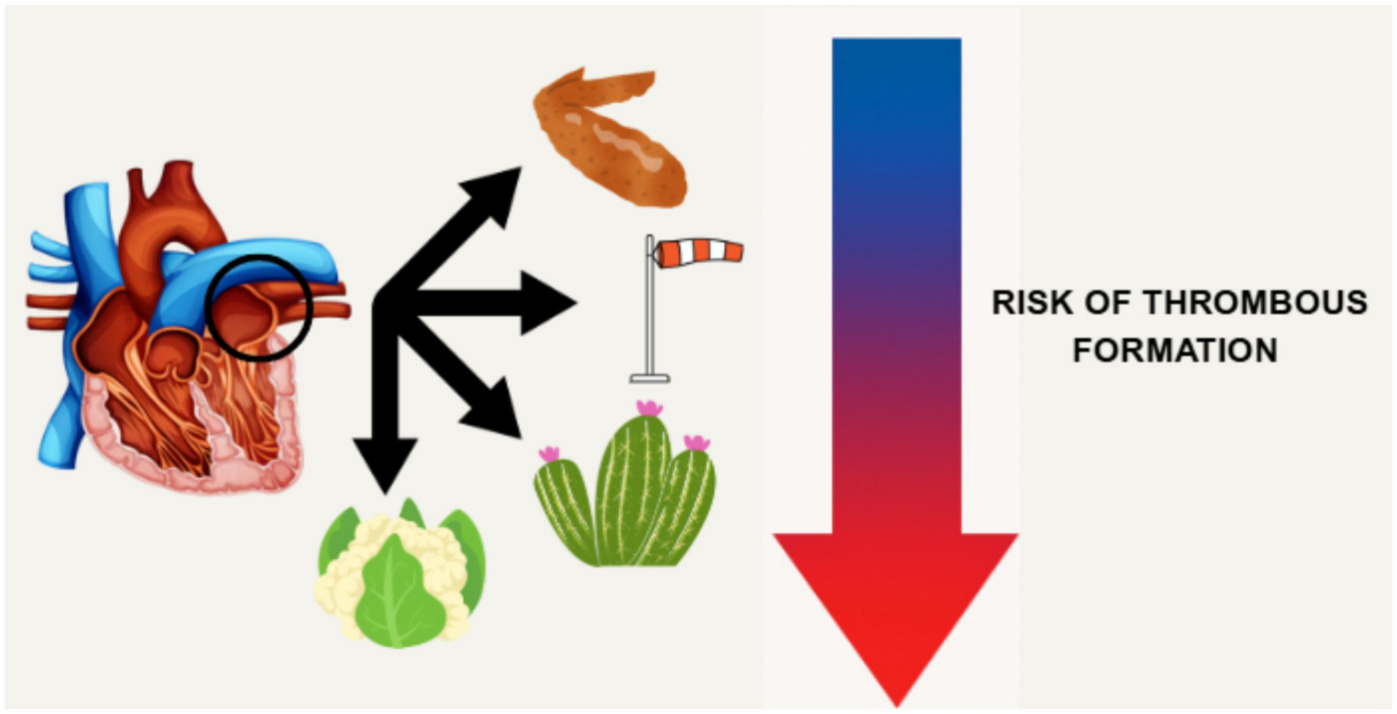
The four common shapes of LAA with associated thrombus formation risk.

The LAA exhibits various morphologies commonly described as chicken wing, windsock, cactus, and cauliflower based on imaging from cardiac CT, MRI, or transesophageal echocardiography. The chicken wing type, marked by a sharp bend in the proximal or middle LAA and it is associated with the lowest risk of thromboembolism and stroke. The windsock morphology features a dominant lobe with variable secondary lobes and carries an intermediate risk, with some evidence suggesting it may be more protective than cauliflower. The cactus form has a central lobe and multiple offshoots. This morphology presents a higher risk of thrombus formation than chicken wing. Finally, the cauliflower type characterized by a short, complex structure with no dominant lobe is linked to the highest risk of thrombus formation and stroke in patients with AF (11, 12). Figure 1 provides a summary of the four kinds of LAA shapes and their associated risk of thrombus formation.

The chicken wing morphology of the LAA can present technical challenges during appendage closure, especially when the bend occurs early and are pronounced. Such cases may limit the depth at which a device can be deployed and often necessitate advanced techniques like the “sandwich” approach or alternative transseptal puncture sites to ensure proper device positioning and sealing. The sandwich technique is an anchoring strategy used when the left atrial appendage has a shallow or challenging landing zone. It involves positioning the Watchman device so that its proximal face is “sandwiched” between the left atrial ridge and the remaining appendage tissue, improving stability and seal when conventional depth-based deployment is not feasible (13, 14). Despite these complexities, procedural success remains high when thorough preprocedural imaging and planning are employed along with careful patient selection. In contrast, the windsock morphology is a relatively straightforward anatomy that facilitates device deployment, anchoring, and sealing. Therefore, it the most common and favorable morphology. This shape is associated with high procedural success and low complication rates (15, 16). Cactus morphology has multiple lobes that can complicate device sizing and positioning. However, the outcomes are generally comparable to other morphologies when appropriate imaging and device selection are utilized (15). The cauliflower morphology presents a short overall length, a complex internal structure, and multiple lobes without a single dominant lobe. These features can make device anchoring and complete sealing more challenging. However, with detailed imaging and careful procedural planning, successful closure is achievable, and success rates are similar to those of other morphologies (15, 16). Procedural success and complication rates for LAA closure are consistently high across chicken wing, windsock, cactus, and cauliflower morphologies when guided by preprocedural imaging and careful patient selection. However, atypical or complex shapes may require advanced techniques and have slightly lower success rates.

## 4 LAA closure procedural techniques

LAA closure is a complex but well-established procedure that follows a series of standardized steps to ensure both safety and efficacy in occluding the LAA. The process begins with comprehensive pre-procedural planning and imaging, where appropriate patient selection is critical. Candidates are typically those with contraindications to long-term oral AC. Imaging modalities such as transesophageal echocardiography (TEE) or Cardiac CT are employed to evaluate the LAA's anatomy. They assist to rule out thrombus, and evaluate accurate device sizing. Once the patient is deemed suitable, the procedure proceeds with vascular access and AC. This is usually performed under general anesthesia or conscious sedation. Venous access is obtained via the right femoral vein, and intravenous heparin is administered to maintain an activated clotting time greater than 250 s, minimizing the risk of thromboembolic complications (17).

The next critical step is the transseptal puncture, which is guided by both fluoroscopy and echocardiography. Puncture is typically made in the posterior and inferior portion of the interatrial septum to optimize alignment with the LAA. Following successful access to the left atrium, a delivery sheath is advanced. This is often done over a guidewire positioned in a pulmonary vein or with the aid of a pigtail catheter. The sheath is de-aired and flushed to prevent air embolism (17, 18). Subsequently, LAA angiography is performed to delineate the appendage's morphology and dimensions. These are essential for selecting the appropriate occlusion device. The device is then prepared and deployed under real-time imaging guidance. It is carefully positioned within the LAA and partially deployed to assess its fit, stability, and seal using TEE and contrast injections (18, 19). Once optimal placement is confirmed, it is important to ensure no significant PDL and a stable position verified by a “tug test”, device is released. Final imaging is conducted to confirm proper deployment and to rule out complications such as pericardial effusion or device embolization (17, 19). The procedure concludes with sheath removal and hemostasis, ensuring that the venous access site is properly sealed to prevent bleeding or hematoma formation. The care is further completed in post op area and most patients able to be discharged home the same day.

## 5 Transesophageal vs. intracardiac echocardiography

Intracardiac echocardiography (ICE) and TEE are two primary imaging modalities used to guide LAA closure. They are each with distinct advantages and limitations. ICE offers several procedural benefits, most notably the ability to perform LAA closure under local anesthesia, which can be particularly advantageous for patients who are poor candidates for general anesthesia. Additionally, ICE is associated with shorter procedural times and reduced fluoroscopy exposure. This can potentially lower the radiation risks for both patients and operators. These features make ICE an attractive option in high-volume centers or in patients requiring minimal sedation (20). However, TEE remains the gold standard in many institutions due to its superior image quality and reproducibility. TEE is especially helpful when visualizing the complex anatomy of the LAA (18). While both ICE and TEE demonstrate similar rates of procedural success and comparable post-procedural outcomes such as stroke prevention and device-related complications, ICE has been associated with a slightly higher incidence of certain complications. These include pericardial effusion, iatrogenic atrial septal defect, and vascular access-related issues. This is mainly due to the need for additional catheter manipulation and venous access (21, 22).

Cost considerations also differ between the two: TEE typically incurs higher costs due to the need for anesthesia personnel, while ICE may involve higher upfront equipment costs but lower overall procedural expenses when all cost is taken into accout. Importantly, most of the current evidence comparing ICE and TEE comes from observational studies and meta-analyses, rather than randomized controlled trials. This limits the strength of the conclusions that can be drawn (23). As technology and operator experience with ICE continue to evolve, its role in LAAC is expected to expand, but careful patient selection and institutional expertise remain key to optimizing outcomes.

## 6 LAA closure device types

The Amplatzer Amulet, Watchman 2.5, and Watchman FLX are the primary devices used for LAA closure, each offering unique design characteristics and clinical advantages. They however maintain comparable overall efficacy and safety profiles. The Amplatzer Amulet features a dual-seal mechanism, consisting of a lobe that anchors within the LAA and a disc that seals the ostium. This design is particularly effective in minimizing PDL and accommodating a wide range of LAA anatomies. In contrast, the Watchman 2.5, the first-generation device, utilizes a single-seal, self-expanding nitinol frame with fixation anchors and a permeable fabric cap. It requires precise sizing and positioning for optimal outcomes. The newer Watchman FLX builds upon the 2.5's foundation with a fully rounded, more flexible frame, additional anchors, and the ability to be fully recaptured and repositioned, which enhances ease of deployment and reduces the risk of complications (24, 25).

In terms of efficacy and safety, all three devices demonstrate high procedural success rates, exceeding 95%. However, differences emerge in specific outcomes. Both the Amulet and Watchman FLX have shown lower rates of significant PDL compared to the Watchman 2.5, with the Amulet offering the best seal due to its dual-seal design. DRT rates are low across all devices, though some studies suggest slightly lower rates with the Amulet and Watchman FLX. In regards to the major adverse events, the Watchman FLX appears to outperform the 2.5. Data has shown reduced rates of complications such as pericardial effusion, major bleeding, and device embolization, and performs similarly or slightly better than the Amulet in this regard. Over the long term outcomes such as stroke, systemic embolism, major bleeding, and mortality are comparable across all devices. There has been no significant differences observed at 1–5 years of follow-up (26–28). From a practical standpoint, the Amulet may involve longer procedural times and a steeper learning curve, particularly for operators unfamiliar with its deployment technique. On the other hand, the Watchman FLX is designed for improved ease of use. This offersa more forgiving deployment process and a favorable safety profile. Importantly, all three devices are FDA-approved for stroke prevention in patients with nonvalvular AF who are not suitable candidates for long-term AC therapy (29, 30).

**Figure 2:**
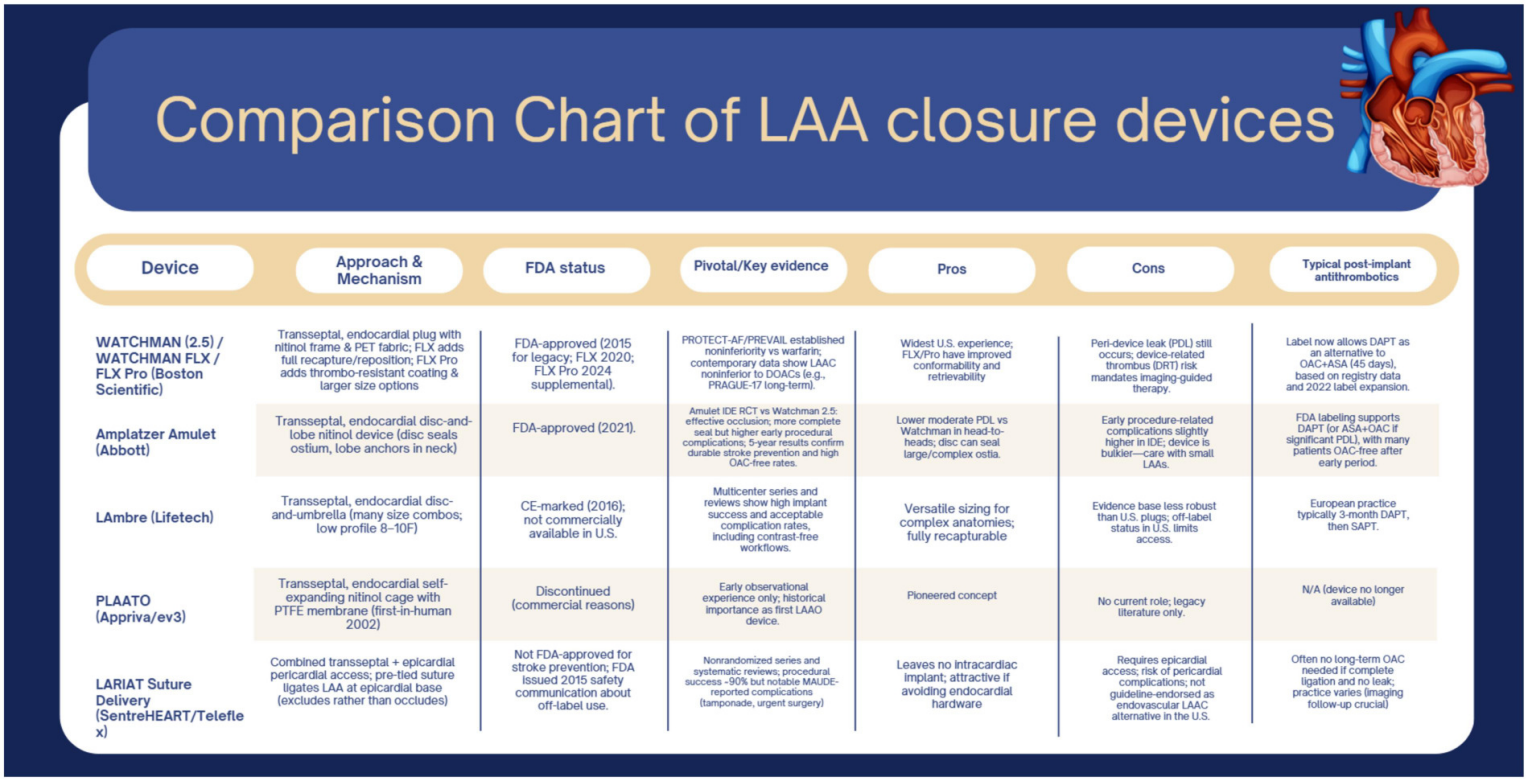
Comparative overview of percutaneous left atrial appendage closure devices.

Recent head-to-head clinical trials, including the Amulet IDE trial and SWISS-APERO, have shown that both the Amplatzer Amulet and Watchman FLX devices offer comparable efficacy and safety LAA closure. However, there are nuanced differences in procedural outcomes and device-specific complications. The Amulet IDE trial, which compared the Amulet device to the Watchman 2.5, found no significant differences in long-term outcomes such as ischemic stroke, systemic embolism, major bleeding, cardiovascular death, or all-cause mortality at both 3 and 5 years. Notably, the annualized ischemic stroke rate was identical at 1.6% per year at the 5-year mark (31, 32). Similarly, the SWISS-APERO trial directly compared the Amulet and Watchman FLX devices and found no statistically significant difference in the composite endpoint of cardiovascular death, stroke, transient ischemic attack, or systemic embolism at 3 years (33). Although the event rate was numerically lower with the Amulet (hazard ratio 0.58; *P* = 0.06), this difference did not reach statistical significance. Per-protocol and as-treated analyses favored the Amulet device, but these findings are considered hypothesis-generating rather than definitive (31–33). Figure 2 summarizes key features, regulatory status, supporting evidence, antithrombotic strategies, advantages, and limitations of currently available and historical percutaneous LAA occlusion/exclusion devices, including WATCHMAN, Amplatzer Amulet, LAmbre, PLAATO, and the LARIAT system.

## 7 Procedural complications

The most commonly observed complications related to LAA closure include pericardial effusion or tamponade, DRT, PDL, vascular access issues, device embolization, and strokes linked to the procedure. Fortunately, these adverse events are relatively rare, and their incidence has decreased over time thanks to advancements in procedural techniques and the development of newer-generation devices.

Pericardial effusion affects about 1%–4% of LAA closure procedures, typically occurring within the first six hours post-implant, though late cases can be seen up to a year later. Most effusions are clinically significant and require pericardiocentesis, with surgical intervention rarely needed (34). Risk is influenced by device type and patient factors, as the double-seal devices like the Amplatzer Amulet carry a higher early effusion risk than single-seal devices such as the Watchman FLX. Female sex, older age, and baseline AC also increase susceptibility (34, 35). Effusions usually result from LAA perforation during device manipulation but may also stem from transseptal puncture or trauma to nearby cardiac structures. Late effusions may be linked to device hooks or persistent inflammation. Outcomes are generally favorable with prompt treatment, though effusions can lead to longer hospital stays and increased costs. Improved operator experience, gentler catheters, and standardized imaging have helped reduce the incidence of serious effusion (18, 34).

Device-related thrombosis (DRT) following LAA closure refers to the formation of thrombus on or near the implanted occlusion device, typically identified through TEE or cardiac CT during routine post-procedural imaging. DRT occurs in approximately 3%–7% of cases, with most events detected within the first 3–6 months, although late occurrences beyond 6 months are possible (18). Several factors increase the risk of DRT. These include a history of stroke or transient ischemic attack, permanent AF, large LAA dimensions, spontaneous echo contrast, vascular disease, reduced left ventricular ejection fraction, older age, and suboptimal device placement. Lower use of oral AC and certain device types may also contribute to risk therefore making patient selection cruicial (36). Clinically, DRT is associated with a significantly increased risk of ischemic stroke and systemic embolism, up to four times higher in affected patients though most strokes after LAAC occur in patients without DRT.Its overall impact on long-term outcomes remains unclear (37). Management typically involves initiating or switching to oral AC, with follow-up imaging at 45–90 day intervals to monitor resolution. While most cases respond well to intensified antithrombotic therapy, persistent or recurrent DRT, especially when larger than 7 mm, may require extended AC. Both TEE and cardiac CT are considered appropriate modalities for ongoing surveillance (18).

Procedure-related stroke LAA closure is uncommon, with ischemic strokes occurring in about 0.1%–0.6% of cases and hemorrhagic strokes being even rarer in most cases. Despite its low incidence, it remains a serious complication requiring prompt recognition and management. Causes include pre-existing thrombus in the LAA, air embolism from inadequate equipment flushing, thrombus formation on catheters or devices due to insufficient AC or prolonged manipulation, and other procedural factors like tissue trauma or excessive device recaptures (18). Risk is elevated in patients with prior stroke or TIA, vascular disease, older age, and higher CHA₂DS₂-VASc scores, as well as in procedures with longer duration, low activated clotting time, or operator inexperience (38). Prevention involves thorough preprocedural imaging, careful equipment handling, and maintaining adequate AC. Continuous neurological monitoring is essential for early detection, and suspected strokes require immediate neuroimaging and stroke team consultation (39). If thrombus is found on equipment, additional heparin may be administered, and the procedure may be paused or aborted. Confirmed strokes are managed using standard acute stroke protocols (18).

PDLs are gaps between the LAA closure device and the LAA wall that allow residual blood flow into the appendage after implantation. These leaks typically result from incomplete sealing due to anatomical variations such as a large or multilobed LAA, elliptical ostium, or suboptimal device sizing and positioning. Factors like insufficient device compression, off-axis deployment, and multiple deployment attempts also contribute. Device design and LAA morphology also key roles in such complications (40). PDLs are relatively common, occurring in 11% to over 50% of cases depending on the device and imaging method used (18). Even small leaks under 5 mm are linked to a modest increase in thromboembolic risk, with larger leaks especially those over 5 mm are associated with a higher incidence of stroke, TIA, and systemic embolism. However, PDLs do not appear to significantly impact cardiovascular or overall mortality (40, 41). Detection is most accurate with TEE or cardiac CT, typically performed 45–90 days post-procedure. TEE remains the standard for grading leak size and guiding management (18, 41). Small leaks are usually managed conservatively, with no clear consensus on restarting oral AC, especially in patients at high bleeding risk. Larger leaks often warrant continued AC if tolerated, and percutaneous closure using coils, plugs, or a second device may be considered. However, in such cases long-term outcomes are still being studied. Repeat imaging is recommended to monitor leak progression, as many resolve over time. This emphasizes the importance of minimizing PDLs during initial implantation (42, 43).

## 8 Procedural optimization

Procedural optimization and innovation in the Watchman LAA closure device have advanced significantly across four domains: device design, imaging, procedural technique, and peri-procedural management. These enhancements have improved safety, efficacy, and patient selection. The next-generation Watchman FLX and FLX Pro devices incorporate key design upgrades over the original Watchman 2.5, including a fully rounded “flex-ball” distal tip. This has enhanced conformability, a broader size matrix such as the 40 mm FLX Pro for larger LAA anatomies and a fluoropolymer coating to improve thromboresistance. These refinements have led to higher procedural success, better LAA sealing, and reduced rates of pericardial effusion, device embolization, and DRT. The FLX Pro expands treatment options for patients with complex or larger LAA anatomy and demonstrates promising early safety and efficacy (44–46).

Imaging and sizing strategies have also evolved, with pre-procedural cardiac CT and intra-procedural TEE now considered standard for optimal device sizing and deployments. CT-based measurements show stronger correlation with final device size and are more effective in minimizing PDL compared to TEE alone. This supports a multimodal imaging approach that enhances procedural planning and outcomes. Procedural techniques have benefited from refined deployment methods, routine use of TEE or intracardiac echocardiography for real-time guidance, and growing operator experience. These factors have contributed to declining complication rates and improved procedural success. Additionally, the FLX device's ability to be recaptured and repositioned with ease further reduces procedural risk (47).

Peri-procedural management has also progressed, with evolving antithrombotic strategies post-implant. Emerging evidence supports the use of dual antiplatelet therapy (DAPT) or direct oral anticoagulants (DOACs) in select patients, offering reduced bleeding risk without compromising protection against DRT or stroke. T these innovations reflect a comprehensive optimization of the Watchman LAAC procedure, enhancing both clinical outcomes and patient safety (48).

## 9 Post-LAA implantation follow up

General management following LAA closure excluding AC and antiplatelet therapy (discussed in the next section) relies on a coordinated approach involving imaging surveillance, patient education, structured clinical follow-up, and multidisciplinary care. Routine post-procedural imaging, most commonly TEE performed around 45 days after implantation, is recommended to evaluate device position, detect PDLs, and identify DRT. Repeat imaging may be warranted if abnormalities are found or if patients develop concerning symptoms (18, 49). Equally important is patient education and vigilant follow-up. Patients should be informed about warning signs of stroke, bleeding, or device-related complications, and monitored for adherence to post-procedure care plans and early indicators of adverse events. For individuals with high bleeding risk or complex comorbidities, multidisciplinary care is strongly advised. Collaborative involvement from cardiology, electrophysiology, and, when appropriate, hematology allows for personalized management and timely intervention should complications arise (50).

## 10 Anticoagulation and antiplatelet management

### 10.1 General population

AC management following Watchman LAA closure is guided by an approach that balances efficacy with bleeding risk along with an individualized approach. The standard regimen begins with warfarin or DOAC combined with aspirin for the first 45 days, transitions to DAPT for up to six months, and then continues with aspirin alone for long-term maintenance. This protocol is based on clinical trials and FDA labeling but should be tailored to individual patient risk profiles and device-specific recommendations. It is important to note that patients can progress from initial AC phase to DAPT after TEE was conducted in follow up and demonstrated no DRT and PDL < 5 mm (18). Emerging data from registries and clinical studies suggest that monotherapy with either DOAC or warfarin excluding aspirin may reduce bleeding risk without increasing thromboembolic events or DRT, and is gaining traction in clinical practice. Notably, both warfarin alone and DOAC alone at discharge have been associated with lower rates of major bleeding and adverse events compared to regimens that include aspirin, without compromising stroke prevention (51, 52). For patients with contraindications to oral AC, DAPT alone for 45 days is now FDA-approved for the Watchman FLX and supported by registry evidence (18). The ADALA trial was a multicenter, randomized clinical study evaluating the safety and efficacy of DAPT vs. low-dose DOAC following Amulet LAA occlusion. Ninety patients were randomized to receive either 3 months of DAPT (aspirin + clopidogrel) or low-dose apixaban (2.5 mg twice daily). The trial found DRT occurred in 8.7% of patients on DAPT compared to 0% in the DOAC group (*P* = .04). Although major bleeding was numerically higher in the DAPT group (13.0% vs. 4.6%), the difference was not statistically significant. No thromboembolic events were reported in either group. These findings suggest that low-dose DOAC may offer superior safety and comparable efficacy to DAPT in patients undergoing LAAC with the Amulet device (53). Despite these options, there remains insufficient evidence to endorse a universally optimal regimen which underscores the need for ongoing trials and individualized risk assessment. Table 1 provides a comprehensive overview of all landmark trials for LAA closure and their AC and antiplatelet regimen. Table 2 highlights the different societal recommendations for LAA closure and their comparison among the expert opinion.

**Table 1:** Summarizes trials and landmark studies for the LAA closure device and AC regimen.

First Author et al., year	Trial name	Trial design	Aim	Key conclusion	Antithrombotic/Anticoagulant Regimen
Goldsweig AM et al., ongoing (68)	CATALYST	Multicenter RCT (Amplatzer Amulet LAAC) vs. DOAC	To evaluate whether LAA occlusion using the Amulet device is non-inferior to DOAC therapy in preventing thromboembolic events and superior in reducing bleeding risks in patients with non-valvular AFat increased risk for stroke.	While final results are not yet published, the trial is designed to determine if device-based stroke prevention can be a safe and effective alternative to long-term anticoagulation with DOAC. The outcome may influence future guidelines on stroke prophylaxis in AF patients.	-
Holmes DR et al., (71)	PROTECT-AF	Multicenter RCT (Watchman 2.5 vs. Warfarin)	Test noninferiority of Watchman vs. warfarin in NVAF	LAAC was noninferior to warfarin; fewer hemorrhagic strokes long-term; initial higher procedural complications.	Warfarin + ASA 45 d → DAPT to 6 months → SAPT
Holmes DR et al., (72, 73)	PREVAIL	Multicenter RCT (Watchman 2.5 vs. Warfarin)	Confirm safety/efficacy vs. warfarin	Improved procedural safety vs. PROTECT-AF; noninferior for ischemic events; pooled data confirmed long-term stroke prevention with less bleeding.	Warfarin + ASA 45 d → DAPT to 6 months → SAPT
Kar S et al., (64)	PINNACLE FLX	Prospective, single-arm (Watchman FLX)	Assess safety/efficacy of FLX device	>98% implant success; low complications; effective closure.	DOAC/Warfarin + ASA 45 d → DAPT → SAPT (per protocol)
Lakkireddy D et al., (32)	Amulet IDE	Multicenter RCT (Amulet vs. Watchman 2.5)	Compare Amulet dual-seal vs. Watchman	Amulet noninferior; fewer leaks; higher early complications.	Warfarin/DOAC + ASA 45 d → DAPT → SAPT (Watchman arm); DAPT upfront allowed (Amulet arm)
Galea et al., (33)	SWISS-APERO	Multicenter RCT (Amulet vs. Watchman FLX)	Direct device comparison	No significant ischemic difference at 3 yrs; numerically fewer events with Amulet.	OAC or DAPT per local practice; both allowed
Reddy VY et al., (74)	ASAP	Prospective, single-arm registry	Evaluate LAAC in OAC-ineligible AF patients	Watchman with DAPT-only feasible and safe; led to ASAP-TOO.	DAPT only (ASA + clopidogrel) for 6 months → SAPT
Reddy VY et al., –ongoing (75)	ASAP-TOO	Multicenter RCT (Watchman vs. medical therapy in OAC-ineligible)	Confirm role of LAAC without OAC	Ongoing; will define DAPT-only regimen efficacy in OAC-ineligible.	DAPT only per protocol
Boersma LV et al., (76)	EWOLUTION	Prospective, multicenter registry (>1,000 pts, real-world)	Evaluate Watchman in clinical practice	High implant success (>98%), flexible antithrombotic regimens; many treated with DAPT or SAPT only.	27% OAC, 60% DAPT, 7% SAPT at discharge; individualized thereafter
Sherwood M et al., ongoing (66)	CHAMPION AF	Prospective, RCT	WATCHMAN FLX LAA closure Device vs. DOACs	To determine whether LAA closure with the WATCHMAN FLX device is a safe and effective first-line alternative to DOAC therapy for stroke prevention in patients with non-valvular AF who are eligible for long-term anticoagulation.	-
Wazni OM et al., (67)	OPTION	Multicenter RCT (Watchman FLX vs. DOAC after AF ablation)	Compare LAAC vs. continued OAC in ablation pts	LAAC reduced long-term bleeding with similar ischemic outcomes.	OAC/DAPT per device protocol vs. DOAC control

**Table 2:** Comparison of guideline recommendations for LAA closure across major societal recommendations.

Clinical consideration	2019 AHA/ACC	2024 AHA/ACC	2025 SCAI/HRS	ESC	EHRA
Primary LAA occlusion indication	May be considered for AF pts at increased stroke risk who have contraindications to long-term OAC (Class IIb)	Reasonable when CHA₂DS₂-VASc is elevated and long-term OAC is contraindicated due to a non-reversible cause (Class IIa).	Supports pLAAC for AF with increased stroke risk and absolute/major contraindication to OAC; provides graded, patient-profiled statements.	Recommended/considered primarily when long-term OAC is not possible (formal contraindication/intolerable bleeding)	Describes who to refer: AF with elevated stroke risk and contraindication to OAC; practical triage & work-up.
Alternative when AC is possible but bleeding risk is high	Not specifically endorsed beyond IIb language	May be reasonable as an alternative to OAC in selected pts with very high bleeding risk (Class IIb).	Addresses shared decision-making for OAC-eligible but high-bleeding-risk pts; selection conditional on individualized risk-benefit.	Generally favors continuing OAC when possible; LAAC as alternative in carefully selected high bleeding risk pts.	Discusses referral in OAC-eligible but high-bleeding-risk scenarios with shared decision-making.
Post-procedure antithrombotic (broad stance)	Not prescriptive; defer to device protocols/trials.	Endorses evidence-based regimens used in trials (e.g., short DOAC + ASA then de-escalation) but leaves specifics to operator/device labeling & evidence.	Provides structured options (e.g., DOAC-based or DAPT-based pathways) tailored to bleeding/thrombotic risk and device imaging findings.	Endorses protocolized antithrombotic strategies per device evidence; emphasizes imaging-guided follow-up.	Practical, device-agnostic pathways (e.g., short DOAC or DAPT, then step-down; TEE/CT-guided adjustments).
Surgical LAA closure (during cardiac surgery)	Not a focus of this update.	Considered when surgery is being done for other reasons; primary emphasis remains on OAC for eligible pts.	Not the focus (transcatheter scope), but aligns with surgical LAA closure when performed concomitantly by other guidelines.	Should be considered as an adjunct to OAC in AF pts already undergoing cardiac surgery (or endoscopic/hybrid AF surgery).	Notes on surgical LAAC exist but focus is on transcatheter referral/follow-up.
Comments	First major U.S. guideline to acknowledge LAA occlusion in AC-contraindicated AF	Reflects upgraded strength of recommendation vs. 2019 based on newer RCT/registry data.	First multi-society U.S. practice guideline dedicated to LAAC; accompanied by a technical review summarizing evidence.	Positions LAAC as niche vs. OAC; clearer positive stance on surgical LAA closure when already in OR.	Consensus/practical guide aimed at everyday clinicians; highly operational.

### 10.2 Coronary artery disease, PCI on DAPT

In patients undergoing Watchman LAA closure who also have a history of percutaneous coronary intervention (PCI) and remain on DAPT, clinical management requires careful balancing of stroke prevention, stent thrombosis risk, and bleeding complications. These individuals face elevated thromboembolic risk due to AF and prior PCI, yet the additive effect of combined antithrombotic therapy significantly increases bleeding potential. The HRS/ACC emphasize individualized antithrombotic strategies post-LAA closure, tailored to device-specific instructions and patient bleeding risk (18). In patients with recent PCI, ACC/AHA guidelines recommend early discontinuation of aspirin, typically within 1–4 weeks and continuation of DOAC plus a P2Y12 inhibitor, preferably clopidogrel, to minimize bleeding risk. For those at high risk of stent thrombosis, aspirin may be extended up to 30 days before transitioning to dual therapy. Evidence from randomized trials and meta-analyses consistently shows that DOAC-based dual therapy yields lower bleeding rates than prolonged triple therapy, without a significant rise in major adverse cardiovascular events (54). Despite these recommendations, real-world practice reveals substantial variability in post-LAA closure regimens. Both DAPT alone and OAC-based strategies are commonly used and appear to offer similar outcomes. Notably, warfarin or DOAC monotherapy excluding aspirin has been associated with reduced bleeding risk and is increasingly adopted in clinical settings. However, most patients do not receive the full protocol outlined in pivotal trials, and further research is needed to clarify optimal antithrombotic combinations and durations for those with concurrent LAA closure and PCI history (54).

### 10.3 Chronic kidney disease and end stage renal disease

Patients with chronic kidney disease (CKD) or end-stage renal disease (ESRD) undergoing Watchman LAA closure often present with AF and face elevated risks of both stroke and bleeding. Risk stratification in this population remains challenging, as the net clinical benefit of AC is uncertain. Traditional scoring systems like CHA₂DS₂-VASc may not reliably identify which dialysis patients derive meaningful protection from therapy. AC management in CKD and ESRD is inherently complex. Current guidelines recommend warfarin or DOACs, especially apixaban for patients with CKD stages 3–5, but careful attention to dosing and adherence to FDA labeling is essential. In patients with ESRD or on dialysis, both warfarin and DOACs carry heightened bleeding risks, and efficacy data are limited due to the exclusion of this population from pivotal DOAC trials (55). Warfarin poses additional concerns in dialysis patients due to labile INR control and potential complications such as calciphylaxis. DAPT serves as a key alternative for patients with contraindications to oral AC. Registry data suggests that DAPT offers comparable safety and efficacy to OAC-based regimens in the post-LAAC setting. The FDA-approved labeling for the Watchman FLX device includes DAPT as an initial regimen, and real-world practice frequently favors DAPT alone in patients with ESRD or high bleeding risk (18, 51).

### 10.4 Left ventricular thrombus

The presence of a concomitant left ventricular thrombus (LVT) introduces in LAA closure presents a layer of complexity. LVT carries a significant risk of systemic embolism, and current guidelines recommend systemic AC for at least 3–6 months, typically with warfarin or increasingly with DOACs (although data in this context are still evolving) (56). Importantly, antiplatelet therapy alone such as aspirin or clopidogrel is insufficient for managing LVT. Therefore, patients with an LV thrombus must remain on AC regardless of Watchman placement. This has direct implications for Watchman strategy. The device is primarily intended to help AF patients avoid long-term AC when bleeding risk is high. Yet, if a patient has another indication for AC such as LVT, a mechanical valve, or recent venous thromboembolism the Watchman does not negate that requirement. In such cases, AC must continue for the duration of the thrombus, and DAPT alone would not provide adequate protection against embolization from the ventricle. Consequently, some centers opt to defer Watchman implantation until the LV thrombus resolves, unless there is a compelling reason to proceed (18, 57, 58).

**Figure 3:**
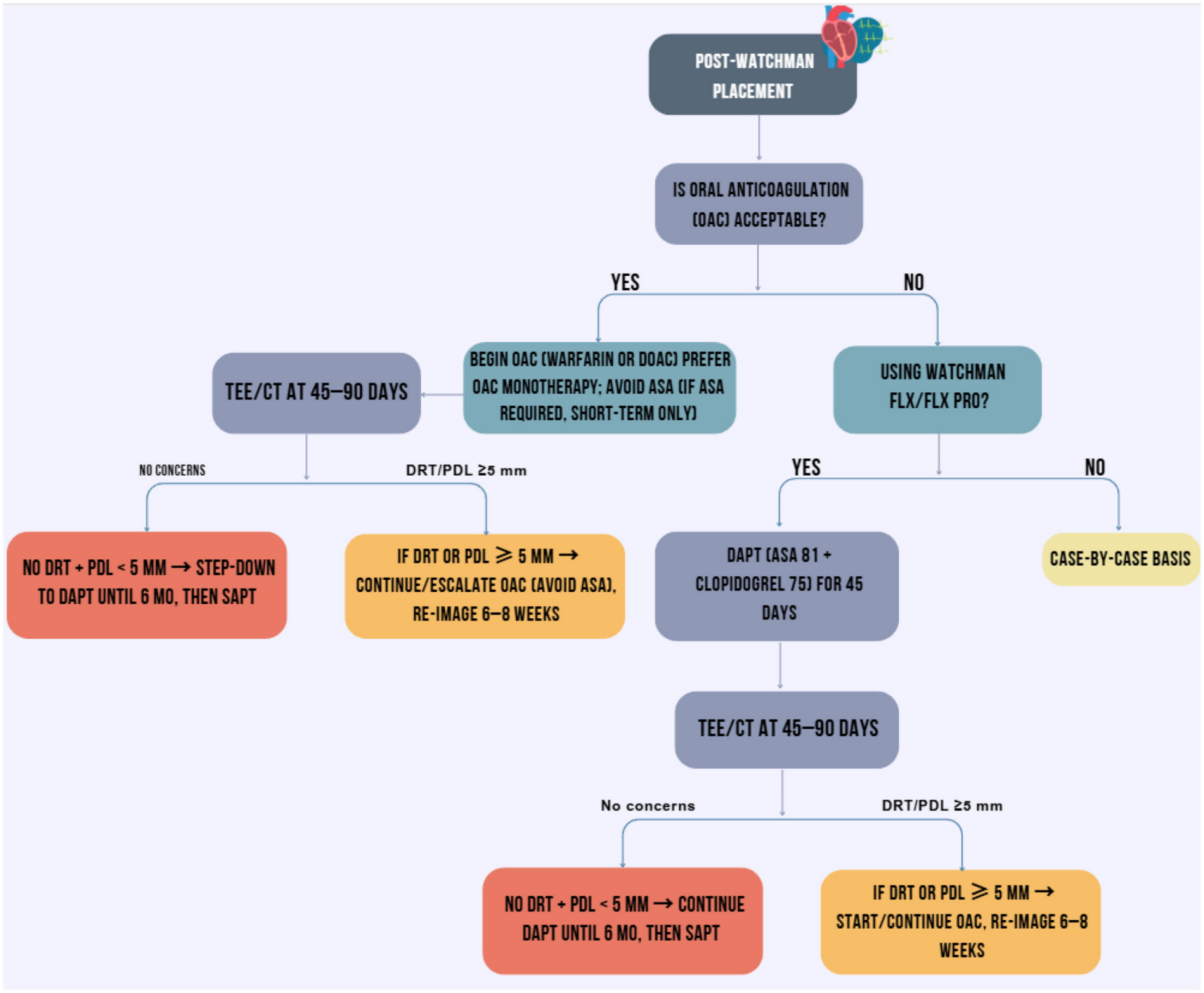
Algorithm for anticoagulation and antiplatelet management after watchman left atrial appendage closure. AF, atrial fibrillation; DAPT, dual antiplatelet therapy; DRT, device-related thrombus; LAAC, left atrial appendage closure; OAC, oral anticoagulation; PCI, percutaneous coronary intervention; PDL, peri-device leak; SAPT, single antiplatelet therapy; TEE, transesophageal echocardiography; CT, cardiac computed tomography.

In practice, if an acute LVT is present at the time of Watchman placement, AC should be maintained for at least 3–6 months, provided renal function permits. Early transition to DAPT should be avoided, as it would leave the thrombus untreated. Once the thrombus resolves, patients with prohibitive bleeding risk may be transitioned to the standard Watchman pathway including DAPT followed by single antiplatelet therapy. If the thrombus recurs or another indication for AC emerges, patients may require indefinite AC despite having the device in place. Figure 3 presents a general approach to AC and antiplatelet management after watchman placement.

## 11 Current challenges

Several persistent challenges continue to shape the landscape of LAA closure using the Watchman platform, despite technological advancements. One major concern is DRT, which remains an unresolved risk even with newer-generation devices like the Watchman FLX. DRT is associated with adverse clinical outcomes, and its management, particularly the choice and duration of antithrombotic therapy varies widely across practices. Recurrent or late-onset DRT has been reported despite seemingly adequate prophylaxis, highlighting the need for more consistent strategies (59). Several large registries and multicenter studies have explored the factors that predict DRT following Watchman implantation. Across these investigations, certain risk factors consistently emerge. These include non-paroxysmal AF (i.e., persistent or permanent forms), a history of stroke or transient ischemic attack, underlying vascular disease, larger LAA diameters, renal insufficiency, hypercoagulable conditions, and deeper device placement (specifically, more than 10 mm from the pulmonary vein limbus) (36, 37). For instance, Simard and colleagues analyzed data from a multicenter registry and identified hypercoagulability, pericardial effusion, renal dysfunction, non-paroxysmal AF, and deep device implantation as independent predictors of DRT. Notably, patients with two or more of these risk factors had a 2.1-fold increased risk of developing thrombus (60). Similarly, Dukkipati et al. found that prior stroke or TIA, permanent AF, vascular disease, larger LAA size, and reduced left ventricular ejection fraction were significant contributors to DRT risk (61).

PDL and residual LAA patency also present diagnostic and prognostic challenges. Although PDL is not uncommon, its clinical significance remains ambiguous. Some studies link PDL to increased thromboembolic risk, but findings differ depending on imaging modality; TEE vs. cardiac CT and across meta-analyses, making it difficult to establish clear thresholds for reintervention or escalation of therapy (40).

Post-implant antithrombotic regimens lack consensus, further complicating care. Current guidelines permit either short-term OAC or DAPT following LAA closure. This reflects alimited comparative evidence. While small, randomized trials suggest that low-dose DOAC may reduce adverse events compared to DAPT, registry data raise concerns about bleeding risk when aspirin is added to DOAC. The optimal agent, dose, and duration particularly in patients with high bleeding risk or contraindications to OAC remain unsettled (42).

The use of the Watchman device in patients with valvular AF presents several significant challenges. One of the primary concerns is the lack of randomized clinical trial data, as major studies evaluating the device have systematically excluded this population (49). Anatomically, valvular AF is often associated with more complex left atrial structures, a higher thrombus burden, and increased procedural difficulty during device deployment. These factors not only complicate implantation but may also elevate the risk of device-related thrombosis. Moreover, patients with valvular AF inherently carry a higher baseline risk of stroke, further complicating risk-benefit assessments. While there are anecdotal reports and limited registry data suggesting feasibility in select cases, robust evidence to support routine use in this subgroup remains lacking, underscoring the need for dedicated studies (62).

Another persistent challenge in LAA closure is achieving accurate device sizing, which is critical for ensuring complete seal and preventing. Optimal sizing depends on precise assessment of the landing-zone diameter, ostial width, and appendage depth measurements. This is typically derived from cardiac CT or multiplane TEE (63). While both modalities are used in practice, CT has consistently demonstrated superior accuracy for defining complex morphologies and correlates more strongly with final device size. For contemporary devices such as the Watchman FLX and FLX Pro, appropriate oversizing generally in the range of 10%–30% relative to the landing-zone diameter is required to achieve stable anchoring and adequate compression (64).

Patient selection poses another layer of complexity, especially when individuals have competing indications for or contraindications to OAC. The benefit of LAA closure is diminished in patients who must remain on long-term OAC for other reasons, such as venous thromboembolism or left ventricular thrombus. Moreover, high-bleeding-risk populations often lack robust randomized data to guide post-implant therapy, a gap underscored by recent multi-society guidance and technical reviews. Finally, operator experience and procedural variability continue to influence outcomes. While large registry data demonstrate high implant success with contemporary devices, results still vary across centers and operators. Decisions regarding device sizing, imaging strategy, and peri-procedural OAC can significantly impact risks such as PDL and DRT, reinforcing the need for ongoing training, procedural standardization, and implementation of quality metrics (65).

## 12 Future direction/ongoing clinical trials

Future directions in LAA closure using the Watchman device are centered on expanding clinical indications, refining post-procedural management, and advancing device technology. A pivotal ongoing trial, CHAMPION-AF (NCT04394546), is directly comparing the Watchman FLX to DOACs as first-line stroke prevention in AF patients eligible for OAC. If noninferiority is established, this could significantly broaden LAAC's role beyond patients with contraindications to O AC (64). Similarly, the OPTION study recently demonstrated that LAA closure performed after AF ablation offers comparable protection against ischemic events and significantly reduces bleeding compared to continued OAC. Many participants underwent LAAC either concurrently with ablation or within 90–180 days, suggesting a potential paradigm shift for rhythm-control strategies (66). Furthermore, the CATALYST trial (NCT04226547) is a prospective, randomized, multicenter clinical study designed to evaluate the safety and effectiveness of the Amplatzer Amulet LAA occluder compared to DOACs in patients with non-valvular AF who are at increased risk for ischemic stroke and are recommended for long-term DOAC therapy. The trial aims to determine whether device-based LAA occlusion can serve as a viable alternative to pharmacologic anticoagulation, potentially reducing bleeding risks while maintaining stroke prevention efficacy. Participants are randomized in a 1:1 ratio to receive either the Amulet device or a DOAC, with the choice of DOAC left to the discretion of the treating physician. The study began in July 2020 and is expected to complete in August 2030, with an estimated enrollment of 2,650 patients (67).

For patients unable to tolerate AC, the ASAP-TOO trial (NCT02928497) is evaluating whether LAA closure combined with antiplatelet-only therapy can safely reduce stroke risk. This study is especially relevant for the highest bleeding-risk populations, where evidence has historically been sparse (68). On the technology front, the Watchman FLX Pro introduces several enhancements, including a fluoropolymer HEMOCOAT surface to accelerate endothelialization, additional radiopaque markers for improved visualization, and a new 40-mm size to accommodate larger appendages. Real-world performance of this next-generation device is being assessed in studies such as HEAL-LAA (NCT05809596) (69).

Post-implant antithrombotic therapy remains an area of active investigation. Although current guidelines permit either oral AC or DAPT following Watchman FLX implantation, clinical practice varies widely. Trials like SIMPLAAFY (NCT06521463) are exploring simplified regimens, including monotherapy, to minimize bleeding risk while maintaining protection against device-related thrombosis. Concurrently, imaging protocols are evolving, with cardiac CT gaining prominence for its ability to detect hypoattenuated thickening and subtle PDL more reliably than TEE. However, standardized thresholds for intervention based on CT findings are still being defined (70).

## 13 Conclusion

LAA closure has matured into a reliable stroke-prevention option for patients with nonvalvular AF who are poor candidates for long-term anticoagulation. Success depends on three pillars: (1) precise anatomic appraisal with CT/TEE to guide device selection and transseptal strategy; (2) disciplined intraprocedural technique that prioritizes coaxial access, appropriate sizing/compression, and rigorous leak/anchoring assessment; and (3) individualized post-implant antithrombotic regimens that balance bleeding and thrombotic risk, particularly in patients with recent PCI or competing indications for anticoagulation. While overall outcomes are favorable across contemporary devices, vigilance for pericardial effusion, DRT, PDL, and rare periprocedural stroke remains essential, supported by structured imaging surveillance and multidisciplinary follow-up. Emerging device designs and coatings, expansion of size options, increasing use of cardiac CT for standardized surveillance, and trials testing simplified pharmacologic pathways are poised to further reduce complications and broaden eligibility especially in post-ablation and AC-contraindicated cohorts. Future priorities include consensus on imaging-driven management thresholds, definitive randomized evidence for de-escalated antithrombotic strategies, and quality metrics that reduce operator variability. As these gaps close, LAA closure will continue to integrate more seamlessly into comprehensive AF care, delivering durable stroke protection with progressively lower procedural and pharmacologic risk.
